# The Unique Associations of Thin‐Ideal Internalization and Internalized Weight Stigma With Body Dissatisfaction Across Body Sizes

**DOI:** 10.1002/jclp.23793

**Published:** 2025-03-26

**Authors:** Emma R. Harris, Samantha L. Hahn, K. Jean Forney

**Affiliations:** ^1^ Department of Psychology Ohio University Athens Ohio USA; ^2^ Central Michigan University College of Medicine Mount Pleasant Michigan USA

**Keywords:** body dissatisfaction, body mass index, sociocultural influences, thin‐ideal internalization, weight stigma

## Abstract

**Objectives:**

The internalization of socioculturally influenced body ideals, including thin‐ideal internalization and internalized weight stigma, is consistently associated with body dissatisfaction. However, the independent contributions of thin‐ideal internalization and internalized weight stigma to body dissatisfaction and the extent to which these two body ideals are distinct are unknown. The current study examined whether internalized weight stigma contributes to body dissatisfaction above and beyond the effects of thin‐ideal internalization. To further investigate the independence of these two cultural processes, the current study also tested if body size moderates the association between thin‐ideal internalization and internalized weight stigma.

**Methods:**

Data come from 430 university students (80.7% female, 87.7% white) who completed surveys for partial course credit.

**Results:**

Both thin‐ideal internalization (*β* = 0.202, *p* < 0.001) and internalized weight stigma (*β* = 0.638, *p* < 0.001) were associated with body dissatisfaction in a mutually adjusted regression model. However, the association between thin‐ideal internalization and internalized weight stigma did not differ by body size (*β* = 0.054, *p* = 0.79).

**Conclusions:**

Thin‐ideal internalization and internalized weight stigma are independent correlates of body dissatisfaction, regardless of weight status. As distinct constructs, both thin‐ideal internalization and internalized weight stigma should be targeted in the prevention and treatment of body dissatisfaction.

## 1 Introduction

Body dissatisfaction, or the negative evaluation of one's body, is common in adolescence and adulthood (Lacroix et al. 2023) and is associated with an increased likelihood of negative psychological outcomes, such as depressive symptoms and disordered eating (Goldschmidt et al. 2016). The internalization of sociocultural influences, including the thin‐ideal and stigmatizing beliefs about weight, has been implicated in the development of body dissatisfaction (Keast et al. 2023; Thompson and Stice 2001). Thin‐ideal internalization (Thompson and Stice 2001) and internalized weight stigma (Puhl et al. 2007) both represent the internalization of societal beauty ideals surrounding shape and weight. Thin‐ideal internalization emphasizes the importance of thinness, whereas internalized weight stigma emphasizes the negative consequences of larger body size. While related, it is unclear to what extent these constructs overlap and/or are distinct. Someone who strongly believes “thin is good” might also strongly hold negative views of overweight status; alternatively, these beliefs may be dissociable. The purpose of this study is to test if thin‐ideal internalization and internalized weight stigma represent distinct constructs and have unique associations with body dissatisfaction.

The Tripartite Influence Model of Body Image and Eating Disturbance (Thompson et al. 1999) posits that the internalization of sociocultural norms mediates the relationship between sociocultural pressures to be thin and body dissatisfaction. Consistent with this model, thin‐ideal internalization predicts body dissatisfaction (Paterna et al. 2021), and changes in thin‐ideal internalization mediate the effects of cognitive‐dissonance‐based body dissatisfaction interventions (Stice et al. 2011). Similarly, internalized weight stigma predicts body dissatisfaction (Keast et al. 2023), and interventions targeting internalized weight stigma are associated with increased body appreciation, a construct closely related to body dissatisfaction (Davies et al. 2022). Given conceptual similarities between thin‐ideal internalization and internalized weight stigma, it is unclear if thin‐ideal internalization and internalized weight stigma are unique risk factors. Indeed, correlations between body ideal internalization and internalized weight stigma have been in the moderate to large range (Lee et al. 2019; Leget et al. 2023). The first aim of this study is to examine the unique associations of thin‐ideal internalization and internalized weight stigma with body dissatisfaction.

If individual differences, such as body size, moderated the relationship between thin‐ideal internalization and internalized weight stigma, this would further support that these are distinct sociocultural constructs. Both thin‐ideal internalization and internalized weight stigma have been positively correlated with body size in adults (Pearl and Puhl 2014; Suisman et al. 2012). The relationship between thin‐ideal internalization and body dissatisfaction is stronger for those with larger body sizes, who deviate more from the sociocultural ideal (Moreno‐Domínguez et al. 2019). Conversely, prior work has demonstrated that the association between internalized weight stigma and body dissatisfaction may not vary based on body size (Romano et al. 2021), suggesting that these sociocultural internalization constructs have unique correlates.

The existing literature suggests that the relationship between thin‐ideal internalization and internalized weight stigma may be stronger for those with larger bodies (Lee et al. 2019). Lee et al. (2019) reported associations between a composite body ideal internalization variable (comprising thin ideal and muscular/athletic ideal), internalized weight stigma, and body satisfaction in a sample of college students stratified by weight status. The underweight/normal subsample tended to have smaller correlations between sociocultural ideal internalization and body satisfaction (*r* = −0.24) and internalized weight stigma and body satisfaction (*r* = −0.57) than the overweight/obese subsample (*r* = −0.35, *r* = −0.73, respectively), although this was not formally tested. Thus, the second aim of this study is to formally test if the relationship between thin‐ideal internalization and internalized weight stigma is stronger for those in larger bodies.

If thin‐ideal internalization and internalized weight stigma are more highly correlated for those in larger bodies, the relative contributions of thin‐ideal internalization and internalized weight stigma to body dissatisfaction may also vary by body size. At larger body sizes, thin‐ideal internalization and internalized weight stigma may overlap more in explaining body dissatisfaction, such that thin‐ideal internalization and internalized weight stigma would not both be uniquely associated with body dissatisfaction. Alternatively, for those with smaller bodies, thin‐ideal internalization and internalized weight stigma may each contribute unique variance to body dissatisfaction. Thus, the final aim of this study is to examine the unique contributions of thin‐ideal internalization and internalized weight stigma, stratified by body size.

Taken together, the present study examined the extent to which thin‐ideal internalization and internalized weight stigma overlap or are distinct constructs. We hypothesized that internalized weight stigma would be associated with body dissatisfaction above and beyond the effects of thin‐ideal internalization. Further, we hypothesized that thin‐ideal internalization and internalized weight stigma would be more strongly associated with those in larger bodies compared to smaller bodies. Finally, we hypothesized that for individuals with smaller bodies, thin‐ideal internalization and internalized weight stigma would each be significantly associated with body dissatisfaction, whereas for those with larger bodies, thin‐ideal internalization and internalized weight stigma would not both exhibit unique associations with body dissatisfaction. Clarifying the unique relationships between thin‐ideal internalization and internalized weight stigma with body dissatisfaction across the weight spectrum will allow more specific intervention targets depending upon individual characteristics.

## 2 Methods

### 2.1 Participants and Procedure

Undergraduate students aged 18 years and older (*N* = 440) were recruited through a departmental research pool at a public American university to participate in an online study from July to November 2020. After providing informed consent, participants completed a questionnaire battery measuring eating and weight‐related attitudes, behaviors, and experiences. Ten participants' data were excluded due to missing significant amounts of data or failing data quality checks (see “Statistical Analyses”) for a final sample size of 430. Participants received partial course credit as compensation. The university's Institutional Review Board approved all procedures.

### 2.2 Measures

#### 2.2.1 Thin‐Ideal Internalization

The 5‐item Thin/Low Body Fat Ideal subscale from The Sociocultural Attitudes Towards Appearance Questionnaire‐4 (SATAQ‐4, Schaefer et al. 2015) measured thin‐ideal internalization. Possible scores range from 1 to 5, with higher scores indicating higher levels of thin‐ideal internalization. Supporting construct validity, the Thin/Low Body Fat Ideal subscale exhibits moderate correlations with eating pathology in United States college students (Schaefer et al. 2015). Supporting discriminant validity, this subscale had small associations with self‐esteem. Internal consistency was good in the current sample (*α* = 0.79).

#### 2.2.2 Internalized Weight Stigma

The 11‐item Modified Weight Bias Internalization Scale (WBIS‐M, Pearl and Puhl 2014) measured internalized weight stigma, independent of weight status. Possible scores range from 1 to 7, and higher scores indicate greater internalized weight stigma. Supporting discriminant validity, the Modified Weight Bias Internalization Scale is only moderately correlated with BMI (Pearl and Puhl 2014). The Modified Weight Bias Internalization Scale had excellent internal consistency in the current sample (*α* = 0.94).

#### 2.2.3 Body Dissatisfaction

The Body Dissatisfaction subscale of the Eating Pathology Symptoms Inventory (EPSI; Forbush et al. 2013)^1^ assessed body dissatisfaction over the prior 4 weeks. Supporting construct validity, the Body Dissatisfaction subscale exhibits large correlations with the Eating Disorder Examination Questionnaire in undergraduate students (Forbush et al. 2014). Eighteen‐day test–retest reliability was good (ICC = 0.64–0.67) in a sample of undergraduate students (Forbush et al. 2019). In the current sample, internal consistency was good (*α* = 0.87).

#### 2.2.4 Body Size

Body mass index (BMI) was used as an index of body size and was calculated using self‐reported height and weight.

#### 2.2.5 Insufficient Effort Responding

The Infrequency Insufficient Effort Responding Scale (Huang et al. 2015) was used to detect insufficient effort responding and ensure participants were attending to the survey items. The eight improbable and/or impossible items that make up the scale (e.g., “I can run 2 miles in 2 min”) were distributed throughout the survey. Participants who endorsed more than four items in the keyed direction, indicating insufficient effort, were excluded from analyses, consistent with recommendations (Curran 2016).

### 2.3 Data Analysis

Four hundred and forty students consented to participate. A total of 10 participants were excluded for the following reasons: providing no data (*n* = 2), failure to complete questionnaires beyond demographic variables (*n* = 1), failing the insufficient effort responding checks (*n* = 6), and reporting an improbable height (*n* = 1), for a final sample of 430. Because missing data was minimal ( < 5% per construct), analyses were conducted using listwise deletion. Linear regression was used to test the unique contributions of thin‐ideal internalization and internalized weight stigma (Hypothesis 1). Body dissatisfaction was the dependent variable, and both thin‐ideal internalization and internalized weight stigma were independent variables in the same regression model. Thin‐ideal internalizaiton was entered in the first step and internalized weight stigma was entered in the second step. To test whether the association between thin‐ideal internalization and internalized weight stigma varied by body size, linear regression was used with internalized weight stigma as the dependent variable and thin‐ideal internalization, BMI, and their interaction as independent variables (Hypothesis 2). Finally, analyses from Hypothesis 1 were repeated in two subsamples: those with a BMI < 25.0 kg/m^2^ and those with a BMI ≥ 25.0 kg/m^2^. To check the assumptions of linear regressions, scatterplots of standardized predicted values and standardized residuals were inspected and were consistent with a linear relationship between variables of interest. A review of Q–Q plots suggested mild heteroscedasticity; as a result, multivariate outliers were removed in sensitivity analyses. The pattern of findings was unchanged. We present results from the entire sample. *α* was set at 0.05.

## 3 Results

The sample was predominately white, female, and heterosexual (see Table 1) and had a mean (SD) age of 21.69 (7.23) years (range 18–54). Table 2 presents descriptive information and correlations for the key variables of interest. BMI was moderately positively correlated with internalized weight stigma and body dissatisfaction but was not associated with thin‐ideal internalization. Both thin‐ideal internalization and internalized weight stigma exhibited large positive correlations with each other and with body dissatisfaction.

**Table 1:** Sociodemographic information of the sample.

	*N*	%
Gender		
Female	347	80.7
Male	76	17.7
Transgender, Genderqueer, or Another Term	7	1.6
Sexual orientation		
Heterosexual	350	81.4
Bisexual	58	13.5
Gay or lesbian	4	0.9
Another term describes me	17	4.0
Missing	1	0.2
Race/ethnicity		
White	377	87.7
Black or African American	28	6.5
Asian	14	3.3
Native Hawaiian or Other Pacific Islander	14	3.3
Hispanic or Latino	12	2.8
American Indian or Alaska Native	2	0.5
Another race/ethnicity	7	1.6

*Note:* For race and ethnicity, individuals were able to select multiple options and were not treated as mutually exclusive.

**Table 2:** Descriptive information and correlations for key variables of interest in a sample of college students.

Variable	*N*	Min.	Max.	Mean	SD	1	2	3
1. Body mass index	410	16.46	53.24	25.38	6.12	—		
2. Thin‐ideal internalization	430	1	5	3.44	0.90	0.01	—	—
3. Internalized weight stigma	430	1	6.82	3.44	1.57	0.44*	0.52*	—
4. Body dissatisfaction	427	0	28	13.78	6.55	0.30*	0.53*	0.74*

* *p* < 0.001.

Table 3 presents a linear regression model testing the independent contribution of internalized weight stigma, above and beyond thin‐ideal internalization, in explaining body dissatisfaction. Both thin‐ideal internalization and internalized weight stigma exhibited unique associations with body dissatisfaction in the mutually adjusted model.

**Table 3:** Hierarchical linear regression testing the unique contributions of internalized weight stigma to body dissatisfaction in a sample of undergraduate students.

	*β*	*t*	*p*
Step 1			
Thin‐ideal internalization	0.531	12.94	< 0.001
Step 2			
Thin‐ideal internalization	0.202	5.51	< 0.001
Internalized weight stigma	0.638	17.39	< 0.001

Next, we tested whether the association between thin‐ideal internalization and internalized weight stigma differed by body size (see Table 4). Both thin‐ideal internalization and BMI exhibited associations with internalized weight stigma in the model. However, the interaction term between BMI and thin‐ideal internalization was not significant, indicating that the association between internalized weight stigma and thin‐ideal internalization did not differ by BMI.

**Table 4:** Linear regression testing body size as a moderator of the relationship between thin‐ideal internalization and internalized weight stigma in a sample of undergraduate students.

	*β*	*t*	*p*
Thin‐ideal internalization	0.471	3.07	0.002
Body mass index	0.400	2.95	0.003
Thin‐ideal internalization × body mass index	0.054	2.68	0.789

Finally, we examined the unique contributions of thin‐ideal internalization and internalized weight stigma to body dissatisfaction among those living in smaller bodies (i.e., BMI < 25.0 kg/m^2^; *n* = 250) and those living in larger bodies (i.e., BMI ≥ 25.0 kg/m^2^; *n* = 158) (see Table 5). The relative effect sizes were similar in both subgroups.

**Table 5:** Linear regression testing the unique contributions of thin‐ideal internalization and internalized weight stigma to body dissatisfaction in a sample of undergraduate students stratified by body size.

	*β*	*t*	*p*
BMI < 25.0 kg/m^2^			
Thin‐ideal internalization	0.196	3.71	< 0.001
Internalized weight stigma	0.587	11.12	< 0.001
BMI ≥ 25.0 kg/m^2^			
Thin‐ideal internalization	0.202	3.48	< 0.001
Internalized weight stigma	0.661	11.38	< 0.001

### 3.1 Sensitivity Analyses

In sensitivity analyses examining the independent contributions of thin‐ideal internalization and internalized weight stigma on body satisfaction that excluded multivariate outliers, results were largely unchanged, and there were no changes to the overall interpretation of findings. However, in sensitivity analyses removing multivariate outliers for the moderation analyses (Hypothesis 2), thin‐ideal internalization (*p* = 0.08) and BMI (*p* = 0.16) were no longer associated with internalized weight stigma. The pattern of results for all analyses was the same once adjusting for gender (coded male, female, and gender expansive), race (coded white, African American/Black, Asian, and another race due to sample cell size), and ethnicity (Hispanic vs. non‐Hispanic).

## 4 Discussion

The present study tested whether thin‐ideal internalization and internalized weight stigma provide unique contributions in predicting body dissatisfaction and if the overlap in constructs differs across the weight spectrum. The current findings suggest that while the two constructs are related, they are uniquely associated with body dissatisfaction. Additionally, the relationship between thin‐ideal internalization and internalized weight stigma did not differ based on body size. Findings suggest that the Tripartite Influence Model of Body Image and Eating Disturbance may consider expansion to include internalization of weight stigma as a contributor to body dissatisfaction (Thompson et al. 1999), similar to expansions that have included internalization of muscular body ideals (Schaefer et al. 2021).

Equifinality posits that there are multiple pathways that can lead to the same outcome (Cicchetti and Rogosch 1996). As a corollary, targeting both thin‐ideal internalization and internalized weight stigma may lead to more effective body image interventions. While cognitive dissonance‐based interventions like the Body Project have demonstrated success in targeting thin‐ideal internalization (Stice et al. 2011), it is unclear if the Body Project influences internalized weight stigma or if it should be augmented to target internalized weight stigma in addition to thin‐ideal internalization. Brief cognitive dissonance interventions targeting weight stigma change explicit, but not implicit, anti‐fat attitudes (Breithaupt et al. 2020). No change in implicit anti‐fat attitudes may mean that more engrained beliefs, such as internalized weight stigma, may not be altered by brief cognitive interventions. One pilot study comparing standard behavioral weight loss to standard behavioral weight loss with the Body Project as an adjunct (Olson et al. 2018) found that internalized weight stigma decreased across time similarly across both conditions. Importantly, this study was limited by a small sample size (*n* = 32) and the emphasis on weight loss. More work is needed to understand if current body dissatisfaction interventions, like the Body Project, are also effective in reducing internalized weight stigma. If augmentation of the Body Project is needed, exercises from existing cognitive behavioral therapy programs for internalized weight stigma (Pearl et al. 2020) might be integrated into future Body Project adaptations.

While the relationship between thin‐ideal internalization and internalized weight stigma did not differ by body size, internalized weight stigma exhibited a moderate correlation with body size, whereas thin‐ideal internalization did not. This differing set of relationships may reflect differences in the volume of stigmatizing experiences based on body size and reinforce the distinct nature of these constructs. The thin ideal may be internalized irrespective of body size, but those with larger bodies may be more likely to internalize weight stigma because they more closely associate their own identity with that sociocultural belief. In addition to developing interventions to help individuals challenge their internalized weight stigma, additional collective work is needed to challenge weight stigma in the sociocultural environment.

The present study had a number of strengths, including the use of well‐described measures, a sample with a wide range of body sizes, and the use of data quality checks. However, results should be interpreted with limitations in mind. The cross‐sectional nature of the work limits causal inferences; longitudinal and experimental work is needed to confirm that both thin‐ideal internalization and internalized weight stigma are risk factors for body dissatisfaction. The current study lacked racial, gender, age, and educational attainment diversity, limiting generalizability, and did not allow us to examine how intersecting identities may impact associations and construct validity. Consistent with prior concerns about measurement overlap between internalized weight stigma and body dissatisfaction (Saunders et al. 2022), we observed a high correlation between internalized weight stigma and body dissatisfaction (*r* = 0.74). Future research is needed in which modified measures of body dissatisfaction and internalized weight stigma are used.

In conclusion, the present study provides evidence that thin‐ideal internalization and internalized weight stigma are distinct constructs that are each associated with body dissatisfaction. Findings suggest that future research should test an expanded Tripartite Influence Model of Body Image and Eating Disturbance that includes internalized weight stigma and investigate whether interventions that target thin‐ideal internalization to reduce body dissatisfaction need to be augmented to also address internalized weight stigma. Findings suggest that the incorporation of weight stigma internalization may increase the efficacy of interventions to prevent and/or reduce body dissatisfaction.

## Conflicts of Interest

The authors declare no conflicts of interest.

## Data Availability

The data that support the findings of this study are available from the corresponding author upon reasonable request.

## Endnotes

1 The instructions of the Eating Pathology Symptoms Inventory were modified to be administered online. From “Development and Validation of the Eating Pathology Symptoms Inventory,” by Forbush, K. T., et al. 2013, Psychological Assessment, 25, 859‐878. Copyright © 2011 by Kelsie T. Forbush. Reproduced with permission. No further reproduction, modification, or distribution of the Eating Pathology Symptoms Inventory, derivative versions, or translated versions is permitted without advance written permission from the copyright holder (Dr. Kelsie Forbush).
